# M. Martin on the Preservation of Cantharides

**Published:** 1842-06-04

**Authors:** 


*The Preservation of Cantharides.*—M. Martin recommends placing the cantharis, either entire or in powder, in rectified sulphuric æther, in the proportion of five parts of the fly to one of æther, in bottles stoppered with emery; and he assures us that the cantharides, thus prepared, can be kept for three years. The æther does not act on it, except as a preservative.—*Provincial Med. Journal, from Bulletin de Thérapeutique.*

